# Sepsis: Current Clinical Practices and New Perspectives: Introduction to the Special Issue

**DOI:** 10.3390/jcm10030443

**Published:** 2021-01-24

**Authors:** Andreas Hecker, Winfried Padberg, Matthias Hecker

**Affiliations:** 1 Department of General & Thoracic Surgery, University Hospital of Giessen, 35392 Giessen, Germany; Winfried.Padberg@chiru.med.uni-giessen.de; 2 Medical Clinic II, University Hospital of Giessen, 35392 Giessen, Germany; Matthias.Hecker@innere.med.uni-giessen.de

Despite modern approaches in intensive care medicine, surgery, and immunology, the mortality of sepsis remains unacceptably high. This Special Issue is dedicated to providing immunologists, intensive care specialists, and surgeons with a platform to publish current modern sepsis research. We are proud to present 14 publications focusing on a broad variety of aspects from basic immunological research to randomized clinical trials on intensive care treatment. Basic research articles were selected concerning their potential to be transferred to clinical medicine in the future. Overall, we have designed an interesting and stimulating scientific Special Issue on modern sepsis epidemiology, diagnosis, and therapy.

Generally, patients suffering from sepsis have a poor prognosis. As an example of patients with intestinal perforation, the survival ranges from 14% (without organ dysfunction) up to 60% (with septic shock) [1]. These data emphasize the importance of performing studies on new approaches and trials, creating scientific evidence for clinicians’ everyday work. Rapid diagnosis is life-saving, and it is essential to provide patients with appropriate therapy based on the three following factors: source control, antimicrobials, and supportive critical care therapy. New diagnostic tools, such as molecular markers (e.g., IL-1β, IL-6, tumor necrosis factor alpha (TNF-α), high mobility group box 1 (HMGB-1) etc.), or DNA-based methods for the detection of specific bacteria aim to accelerate the diagnostic pathways but still require further evaluation. The increasing understanding of the innate immune response in the early phase of Systemic inflammatory response syndrome (SIRS) and sepsis assists not only in the design of new diagnostic tools, but also in realizing the potential of an early pharmacological intervention. In this Special Issue, we aim to provide an update on modern clinical and immunological research on this important field of clinical everyday work. Overall, this Special Issue presents a collection of 14 scientific articles dealing with new epidemiological, diagnostic, or therapeutic approaches on sepsis and systemic hyperinflammation generally reflecting the whole spectrum of research from basic science through translational research to clinical and epidemiological studies.

## 1. Epidemiology of Sepsis (Three Publications)

One population-based analysis of a Spanish hospital discharge database published in this Special Issue revealed that, even in the young study population (aged 20–44 years), the in-hospital sepsis mortality was 24% (2006–2015) and associated with age, comorbidity, and the extent of organ dysfunction [2]. One metabolic disease with a dramatically increasing prevalence in the first world is obesity, which was analyzed in regard to its impact on sepsis survival by Mewes et al. [3]. Despite the negative long-term effects of obesity, such as the development of arterial hypertension, diabetes mellitus, etc., interestingly, there seems to be a protective effect of obesity on sepsis survival. Obese patients (body mass index > 30 kg/m^2^) showed decreased 90-day mortality in the Caucasian study group [3].

The detailed analysis of the Spanish hospital discharge database, mentioned above, further emphasizes the importance of the respiratory tract as the major source of septic infections (about one-third) [2]. Genitourinary tract infections are the second most common source of sepsis in young adults [2]. In this Special Issue, Patel et al. published an interesting epidemiological study on the incidence of urinary tract infections, which are thought to be more common in children with sickle cell anemia. Interestingly, the authors were not able to confirm this hypothesis by analysis of urinary samples of 167 febrile children (<4 years of age) with sickle cell anemia. With 4.1% of urinary infections, the rate of urinary tract infections was as high as in non-sickle cell anemia children [4].

## 2. New Diagnostic and Immunological Approaches (Seven Publications)

Two publications by Schneck et al. analyze the method of flow cytometry-based quantification of neutrophil extracellular traps (NETs) as a potential novel diagnostic tool in sepsis and in the perioperative phase. NETs correlated positively with FIBTEM mean clot firmness (MCF) in septic shock patients, while they correlated negatively in surgical patients [5]. Stimulated by this first publication, Dr. Schneck and his team were invited to publish a second analysis of their study collective. The large amounts of blood samples and clinical data were evaluated in regard to further potential serum markers, thereby allowing a distinction between sepsis and postsurgical systemic hyperinflammation. This, of course, would be of major clinical importance for surgeons and intensivists to detect septic complications in the postoperative phase more rapidly. NADH dehydrogenase 1(ND1) mitochondrial DNA (mtDNA) was discriminative between sepsis and surgery-induced hyperinflammation; thus, it might serve as an interesting new diagnostic target for intensive care medicine [6].

Rapid diagnosis of sepsis is of major importance and is lifesaving. Early serum markers, such as IL-6, IL-1α, tumor necrosis factor alpha (TNF-α), high mobility group box 1 (HMGB-1), matrix metalloproteinase 9 (MMP-9), vascular endothelial growth factor (VEGF), intercellular adhesion molecule 1 (ICAM-1), myeloperoxidase (MPO), methylglyoxal, and caspase-3 have been identified as sensitive indicators of sepsis development [1]. As published by Morris et al., exosome proteomic profiling could be another approach to distinguish between sick and healthy in the very early phase of sepsis. Patient samples collected in the emergency department (septic patients *n* = 7 vs. healthy control group, *n* = 5) were analyzed in regard to the exosome profile. Interestingly, 62 proteins differed between the exosomes of septic vs. healthy subjects. Thus, in this Special Issue, the authors present a novel diagnostic approach which could be of importance for further clinical trials [7].

As published by the group of Dr. Veronika Grau, C-reactive protein (CRP) has an anti-inflammatory impact on the ATP-mediated assembly of the NLRP3 inflammasome in monocytes [8]. The activation of the NLRP3 inflammasome is an essential step in the synthesis of mature IL-1β; thus, it plays a key role in innate immunity. We are proud to present a clinical study of the same group, offering the first findings that elevated preoperative CRP-levels might also be anti-inflammatory and could attenuate the postoperative increase in inflammatory markers [9]. As a further key player for ATP-mediated monocytic IL-1β release, amyloid-β (1-42) peptide seems to be a modulator of the cholinergic inhibition of the NLRP3 inflammasome. It has the capacity to neutralize the anti-inflammatory cholinergic effect, thus enabling IL-1β release, although cholinergic inhibitors are present in vitro [10]. In contrast to the potential beneficial effect of a pre-existing CRP elevation, upregulation of CRP in cases of an existing pathological state (e.g., intracerebral hemorrhage) seems to have a negative impact on patients’ survival. Bender et al. analyzed patients with spontaneous intracerebral hemorrhage and were able to identify a CRP/albumin ratio > 1.22 on admission as an predictor for in-hospital mortality [11]. 

While anti-inflammatory mechanisms could be lifesaving in the early hyperinflammatory phase of sepsis, later on, the patient is threatened by prolonged immunosuppression and immunoparalysis, called compensatory anti-inflammatory response syndrome (CARS). Moreover, apoptotic mechanisms resulting in T cell depletion play a major role in the pathogenesis of CARS; Sommer et al. were able to demonstrate that decreased thymic output and increased aging of CD4^+^ T cells may additionally contribute to lymphopenia. This mechanism could allow the development of new pharmacological approaches in the future [12].

## 3. Sepsis Recognition and Therapy (Three Publications)

Clinical data published by Reichert et al. reveal that both the minimally invasive hybrid esophagectomy and the open approach bear the risk of respiratory impairment and pneumonia development in the postoperative phase. Interestingly, the minimally invasive technique is associated with equal rates of pulmonary impairment, but has a significantly lower risk of pneumonia after surgery [13]. Once respiratory impairment occurs, patients often require parenteral nutrition after major surgery. Although potential positive immunomodulatory effects of parenteral nutrition are intensively discussed in the literature, Hecker et al. were able to show that the partial replacement of n-6 fatty acids by n-3/n-9 fatty acids (SMOF, soybean oil, medium-chain triglycerides, olive oil, and fish oil) showed beneficial anti-inflammatory and resolving effects in murine acute respiratory distress syndrome (ARDS). Mice infused with SMOF showed decreased leukocyte invasion, protein leakage, myeloperoxidase activity, and cytokine production in alveolar spaces after LPS challenge compared to animals that received pure long-chain triglyceride solution. Further studies will be necessary to confirm these data. Nevertheless, the importance of balanced nutrition for critically ill patients in the post-surgical phase will be an important field of research in the future [14]. Besides the immunomodulating effects of nutrition, adjunctive pharmaceutical therapeutic approaches are controversially discussed both in sterile SIRS (e.g., after surgery) and in sepsis. For septic patients, Park et al. analyzed the impact of early vitamin C and thiamine administration on ICU delirium-free days among critically ill patients in septic shock. Propensity score-matched pair analysis between the treatment and control group suggested that no beneficial effects of vitamin C or thiamine administration could be detected regarding delirium-free days [15]. 

## 4. “Sepsis-Associated Encephalopathy”: A Review on the Pathogenesis and Clinical Presentation

One review deals with the important topic of “sepsis-associated encephalopathy”. While sepsis-associated encephalopathy in the acute phase of sepsis leads to increased mortality rates, it dramatically reduces the quality of life of patients surviving septic diseases later on. Chung and colleagues present an interesting review on both the clinical aspects and pathomechanisms contributing to sepsis-associated encephalopathy in an excellent manner [16].
